# A simulation study investigating potential diffusion-based MRI signatures of microstrokes

**DOI:** 10.1038/s41598-021-93503-2

**Published:** 2021-07-09

**Authors:** Rafat Damseh, Yuankang Lu, Xuecong Lu, Cong Zhang, Paul J. Marchand, Denis Corbin, Philippe Pouliot, Farida Cheriet, Frederic Lesage

**Affiliations:** 1 Laboratory of Optical and Molecular Imaging, École Polytechnique de Montréal, 2900 Edouard Montpetit Blvd, Montreal, QC H3T 1J4 Canada; 2 Athinoula A. Martinos Center, Massachusetts General Hospital, Harvard Medical School, Charlestown, MA 02129 USA; 3 Montreal Heart Institute, 5000 Rue Bélanger, Montreal, QC H1T 1C8 Canada; 4 Université de Montreal, 2900 Edouard Montpetit Blvd, Montreal, QC H3T 1J4 Canada; 5 Department of Computer and Software Engineering, École Polytechnique de Montréal, 2900 Edouard Montpetit Blvd, Montreal, QC H3T 1J4 Canada

**Keywords:** Computational biology and bioinformatics, Biomarkers, Engineering, Mathematics and computing

## Abstract

Recent studies suggested that cerebrovascular micro-occlusions, i.e. microstokes, could lead to ischemic tissue infarctions and cognitive deficits. Due to their small size, identifying measurable biomarkers of these microvascular lesions remains a major challenge. This work aims to simulate potential MRI signatures combining arterial spin labeling (ASL) and multi-directional diffusion-weighted imaging (DWI). Driving our hypothesis are recent observations demonstrating a radial reorientation of microvasculature around the micro-infarction locus during recovery in mice. Synthetic capillary beds, randomly- and radially-oriented, and optical coherence tomography (OCT) angiograms, acquired in the barrel cortex of mice (n = 5) before and after inducing targeted photothrombosis, were analyzed. Computational vascular graphs combined with a 3D Monte-Carlo simulator were used to characterize the magnetic resonance (MR) response, encompassing the effects of magnetic field perturbations caused by deoxyhemoglobin, and the advection and diffusion of the nuclear spins. We quantified the minimal intravoxel signal loss ratio when applying multiple gradient directions, at varying sequence parameters with and without ASL. With ASL, our results demonstrate a significant difference (p < 0.05) between the signal-ratios computed at baseline and 3 weeks after photothrombosis. The statistical power further increased (p < 0.005) using angiograms measured at week 4. Without ASL, no reliable signal change was found. We found that higher ratios, and accordingly improved significance, were achieved at lower magnetic field strengths (e.g., B0 = 3T) and shorter echo time TE (< 16 ms). Our simulations suggest that microstrokes might be characterized through ASL-DWI sequence, providing necessary insights for posterior experimental validations, and ultimately, future translational trials.

## Introduction

Cortical microvascular networks are the carrier of continuous supply of oxygen and energy substrates to neurons, and thus they are responsible for maintaining their healthy state. These networks react dynamically to meet the rapid and substantial increases in energy demands during neuronal activation through the process of neurovascular coupling^1^. Structural deterioration of the cortex microvasculature directly disrupts the regulation of cerebral blood flow and alters the distribution of oxygen and nutrients. Among pathogenic outcomes in cerebrovascular diseases^2^ is the emergence of micro occlusions in penetrating arterioles descending from the pial surface. Recent experiments have provided evidence about the impact of these microscopic events on brain function^3^. Occlusion of a single penetrating vessel was shown to lead to ischemic infarction in the cortex^4^ and to have effects on targeted cognitive tasks. Cerebral microinfarcts have emerged as a potential determinant of cognitive decline, as they are one of the most wide-spread forms of tissue infarction in the aging brain^5^. These cortical lesions have been associated to severe deficits in motor output at muscles^6^. It was also shown that a microembolism of a single cortical arteriole induces cortical spreading depression, a potential trigger and putative cause of migraine with aura^7^. In a separate study, the induction of microvascular lesions in an Alzheimer’s mouse model was shown to alter both the deposition and clearance of amyloid-beta plaques. Optical microscopy and photoacoustic imaging are potential techniques for imaging the local architecture of cerebrovascular morphology at micro-scale, however, they remain invasive and are currently limited to preclinical studies. Given the strong association between these microvascular events and many neurological disorders, developing non-invasive and translatable approaches is of vital importance to identify their presence in clinical settings.

A recent study based on 2-photon microscopy has illustrated that the capillary bed in microvascular networks regenerates into a radially organized structure following a localized photothrombotic infarction^8^. To overcome limitations due to fluorescent dye leakage through the damaged blood–brain barrier, recent work exploiting optical coherence tomography (OCT) provided a detailed exploration of the microvascular angio-architecture rearrangement at different cortical depths^9^ following photo-thrombosis. This latter study confirmed the presence of highly radially organized patterns, at all cortical depths, with a higher degree of structural reorganization in deeper regions. These morphological features could be exploited as clinical signatures of the associated ischemic events.

In this study, we hypothesize that these vascular re-orientations can be detected through magnetic resonance imaging (MRI), and we provide a proof-of-concept through simulations. Our assumption is based on diffusion-weighted imaging (DWI), which is an established MRI technique that provide contrast sensitive to the motion of water molecules^10^. DWI is widely applied to capture white matter tracts^11^, through the use of arbitrarily selected directions of the diffusion gradient to measure the directional bias of molecular movements. A sub-type of DWI is the intra-voxel incoherent motion (IVIM) technique, used to detect the high pseudo-diffusion coefficient that is attributed to the vascular component of the tissue^12^. Pseudo-diffusion measurements were used to quantify changes in cerebral perfusion^13^. Previous studies have shown that a hybrid scheme, combining IVIM and multi-direction DWI, could allow for a measurable effect size caused by microcirculation architecture^14–17^. Phantom-based simulations and realistic measurements of calf muscle were performed to characterize capillary anisotropy of skeletal muscle microvasculature^14^. Another study obtained similar measurements in the placenta^16^. Moreover, a further in-vivo study used the same approach to characterize microvascular renal flow anisotropy^15^. Arterial spin labeling (ASL) was combined with an IVIM model to show that such technique is able to capture the dominant directionality of cerebral microvasculature in the rat brain^17^. Here, we conduct IVIM spin echo realistic simulations, to investigate potential signatures of cerebrovascular micro-occlusions induced in mice brains after targeted photothrombosis. Taking advantage of the radial angiogenesis around the micro-infarction locus after occlusion, we propose a measurable biomarker based on quantifying the ratio of directional signal loss induced when using multiple gradient directions. By integrating and excluding ASL, we performed parametric simulations using different field strengths, echo time TE, *b*-values and gradient duration. This study provides meaningful insights about the effect size as a function of parameters, which could be of great benefit for guiding further experimental investigations.

## Results

### Experimental design

**Figure 1:**
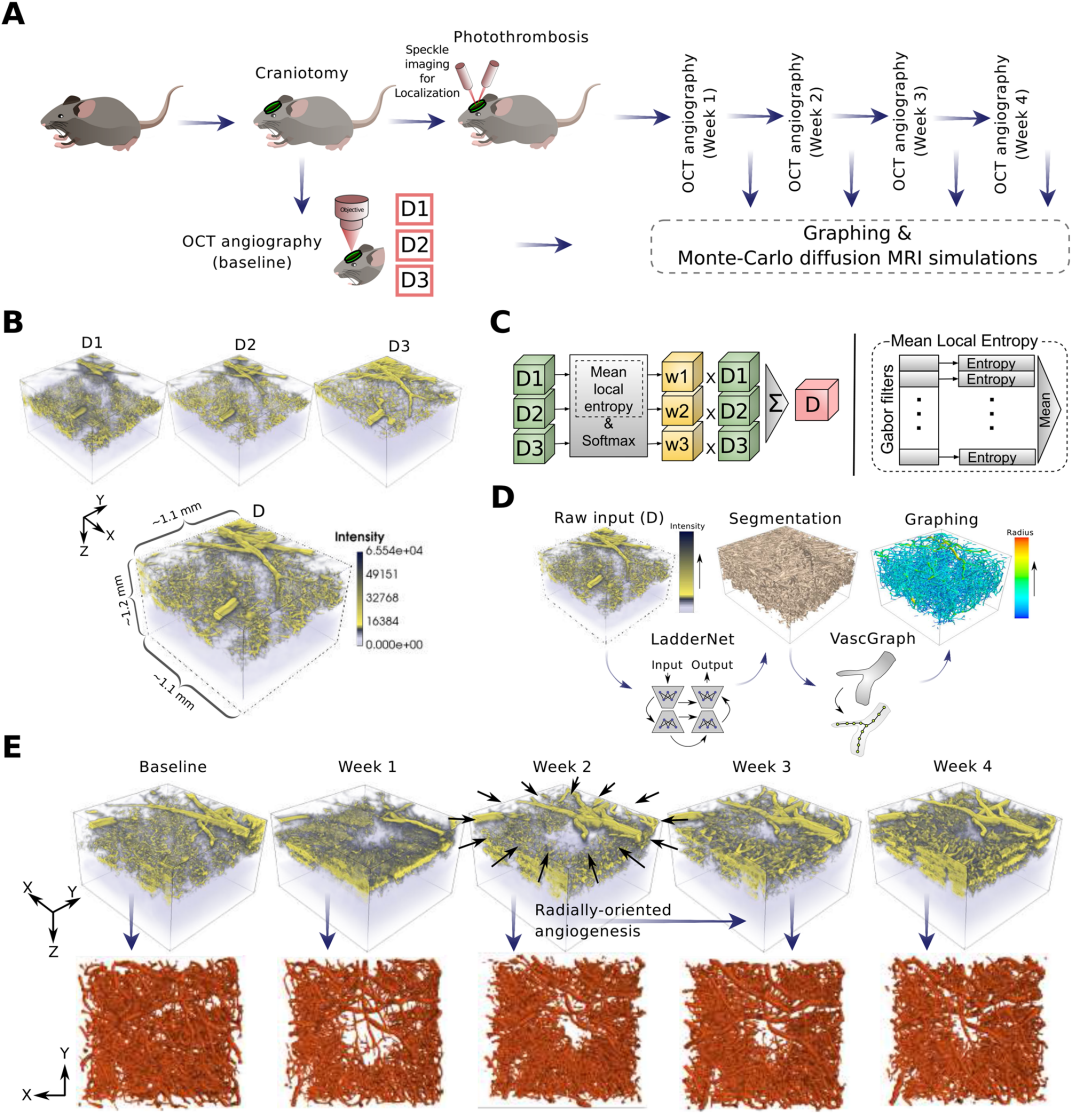
(**A**) Our experimental procedure for inducing and monitoring of micro-occlusions. Depth-dependant Pre- and post-lesion OCT angiographic acquisitions were preformed to capture vascular degeneration. The OCT stacks acquired at different time points are fed to our computational pipeline to study differences in their diffusion MRI response. (**B**) Reconstruction of a final 3D OCT angiogram *D* from three depth-dependent images, namely, D1, D2 and D3. (**C**) Our technique of reconstructing D is based on taking the weighted sum of D1, D2 and D3. The associated weights are determined by the mean local entropy calculated from D1, D2 and D3 after processing them with a set of Gabor filters. The image with richer vascular structures contributes more to the weighted sum. (**D**) Our image processing pipeline used to extract useful structural/topological models of vascular networks. These models are essential to perform our Monte-Carlo MRI simulations. The segmentation is based on a customly trained LadderNet architecture. We used The VascGraph toolbox^21^ to obtain graph-based vascular skeletons that can approximate the needed anatomical information. (**E**) 3D rendering of the vascular structure before and after creating a photothrombotic lesion. A noticeable radial-wise orientation is observed after-lesion especially following Week 2. The mouse image in (**A**) is reproduced from^55^.

**Figure 2:**
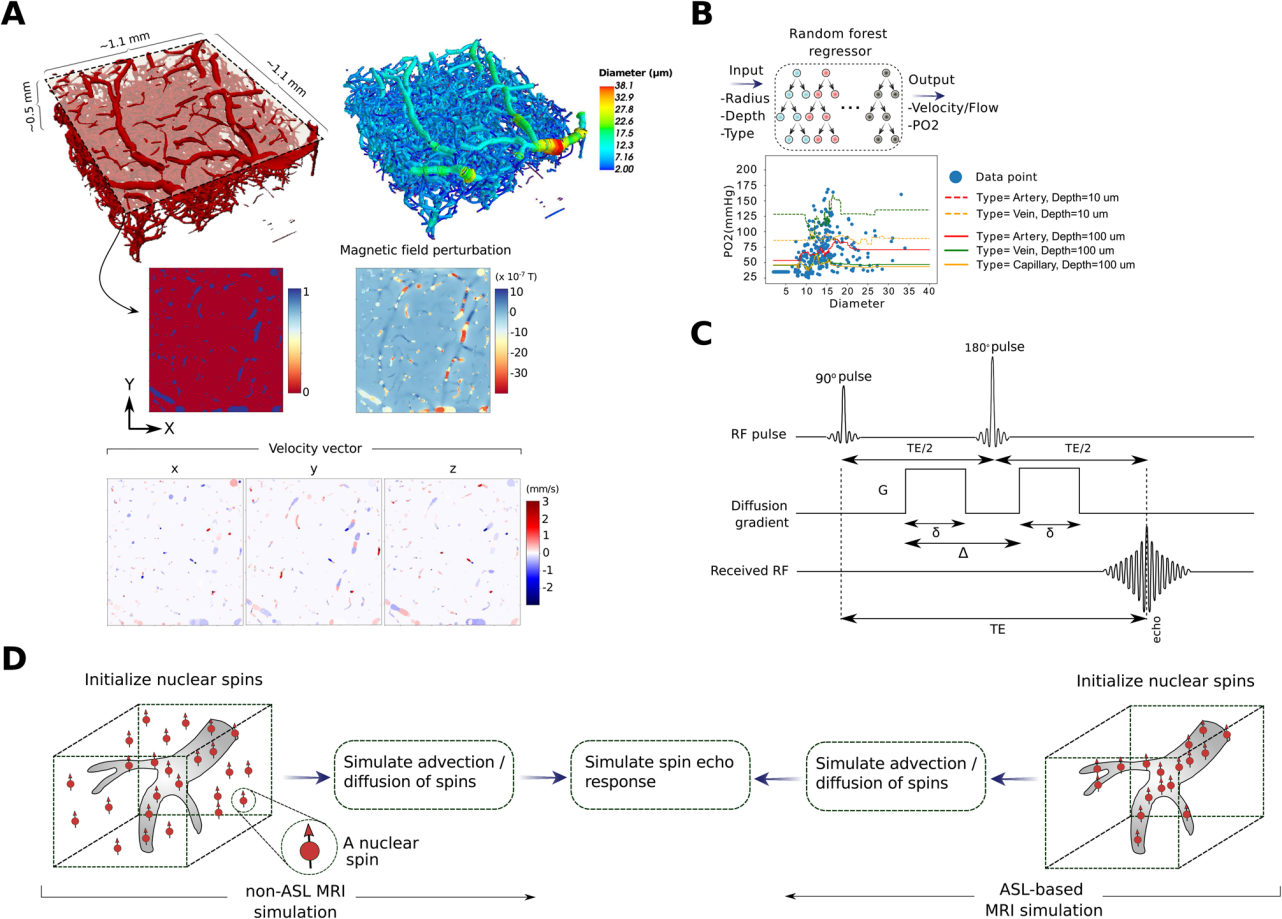
The simulation framework used to compute the MRI response through the diffusion and advection of nuclear spins within the cerebral microvasculature. (**A**) The computation of alterations in the main magnetic field due to the distribution of deoxyhemoglobin in the blood. (**B**) Approximation of PO2 values using our random forest model; these predictions are essential in order to compute the magnetic field perturbations. Following the same machine learning approach, we estimated the velocity/flow field that drives the advection of the spins in our MRI simulation scheme. (**C**) The DWI sequence used in our MRI simulations for each gradient direction. (**D**) The difference between the non-ASL and ASL-based approaches used to simulate the DWI spin echo response in our work.

A demonstration of the experimental workflow followed to test our hypothesis is depicted in Fig. 1A. We used a photothrombotic model to induce a localized micro-occlusion of an ascending arteriole in the barrel cortex of mice. We obtained pre- and post-lesion OCT angiographies to capture the associated vascular degeneration. These OCT acquisitions were acquired before occlusion (baseline) and at weeks 1, 2, 3 and 4 after the occlusion to allow longitudinal monitoring of vascular parameters. As described in Fig. 1B, to construct one OCT angiogram (stack), denoted as *D*, three depth-dependent acquisitions, namely, *D*1, *D*2, and *D*3 were obtained from the same region by adjusting the depth of the imaging focal point. The image *D* was computed as the weighted sum of the three depth-dependant images as depicted in Fig. 1C. The weights were assigned by applying a softmax function to the mean local entropy calculations from the three images^18^. More details about computing the mean local entropy from each image is provided in “Methods” section. Pre- and post-lesion OCT reconstructed angiograms were fed to our image/geometry processing pipeline to generate computational graphs of the contained vascular networks, see Fig. 1D. First, we segmented the vascular structures from OCT stacks using LadderNet^19^, which is a special type of convolutional neural networks trained on our in-house labeled data. Segmentation outputs were then processed with the VascGraph toolbox^20,21^ to produce the final graphical models of the microvasculature. Taking the advantage of the radial angiogenesis around the micro-infarction locus after occlusion as seen in Fig. 1E, we exploited such vascular models to derive potential MRI biomarkers. The produced vascular anatomical models were used as inputs to our Monte-carlo simulation framework to study the associated diffusion MRI response, see Fig. 2. To enable the study of the MRI response, we predicted necessary physiological parameters, namely, velocity/flow and partial pressure of oxygen (PO2) on vascular segments based on their geometric features, see Fig. 2B. Our predictor is a random forest model trained on experimental measurements and it is thoroughly explained in “Methods” section. We used these physiological approximations to calculate the magnetic field perturbations and other MRI parameters. Finally, we simulated the advection and diffusion of millions of nuclear spins following the (spin echo) DWI sequence depicted in Fig. 2C, using various gradient directions and parametric setups. The parameters that specify the DWI experiments were the main field strength B0, the echo time TE and the *b* value, which is dependent on the strength of the two gradient pulses, *G*, the time duration of each one, $\delta$, and the time separation between them, $\Delta$. In order to search for potential signatures of microstokes in our work, we used two approaches for simulating the DWI spin echo responses, namely, non-ASL and ASL-based; see Fig. 2D. In non-ASL simulations, we computed signal contributions from nuclear spins initialized to cover the entire neurovascular unit. On the other hand, in an ASL-based experiment, we computed the accumulated signal originating only from the intravascular space with no nuclear spins initialized in tissue. Our ASL-based simulations mimic the response of an experimental ASL MR imaging, where the arterial blood is tagged so one can observe only the signal that is produced from spins in the blood in the slice of interest.

### Differences between diffusion MRI responses due to altered vascular orientations

**Figure 3:**
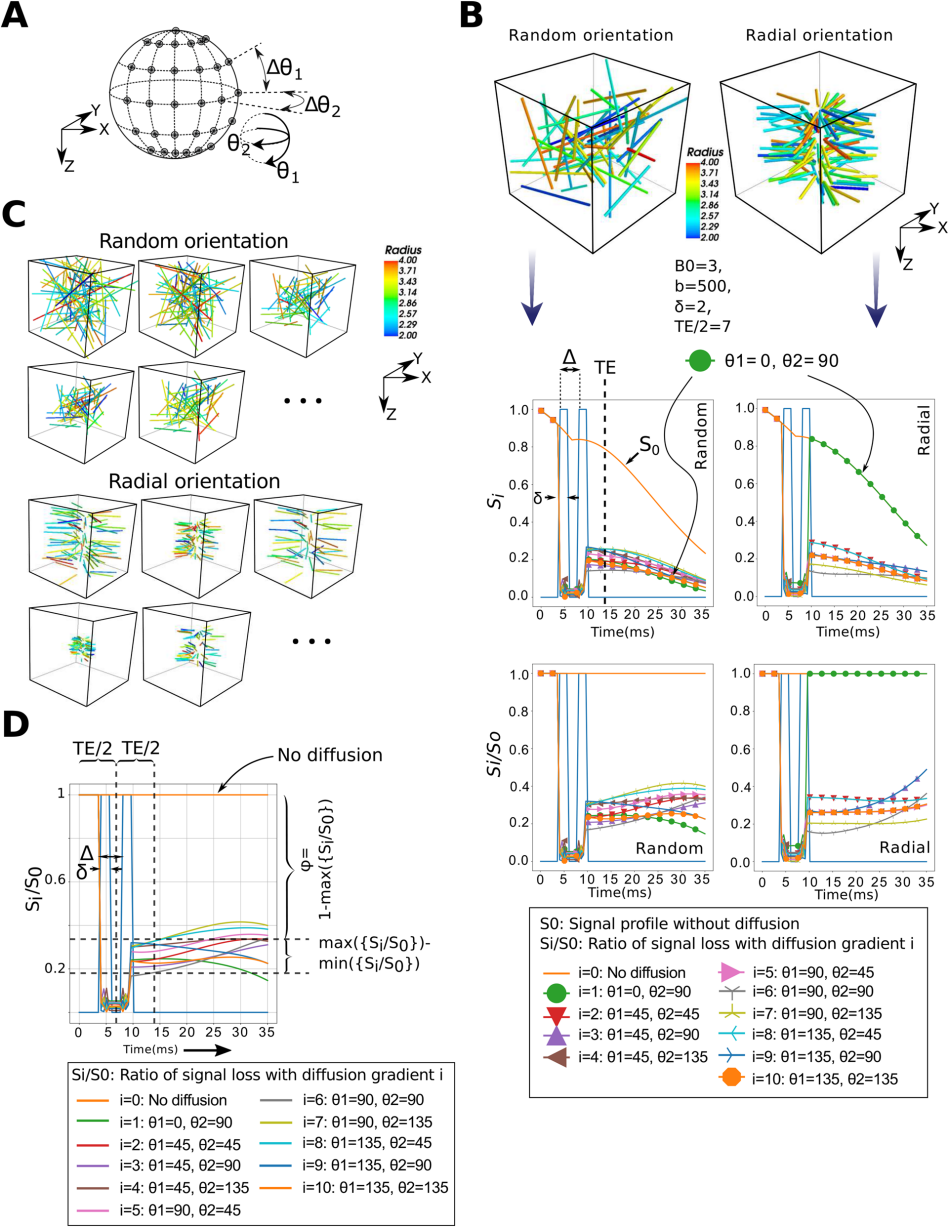
(**A**) A depiction of the different but isotropically distributed gradient directions used in DWI simulations. These gradients are controlled by $\Delta \theta _1$ and $\Delta \theta _2$. (**B**) The difference between two diffusion-based MRI responses simulated-using our Monte-Carlo framework-from synthetic randomly- and radially-oriented capillary structures. For each sample, we simulate signals resulting from using different gradient directions. (**C**) Examples from the two groups in our synthetic dataset. (**D**) The quantification of signal variations after simulating the diffusion MRI responses of a vascular unit using different gradient directions.

Exploiting Monte-Carlo MRI simulations, on synthetically approximated capillary beds, we show that orientations of vascular geometry lead to different profiles of the diffusion MRI signal. We constructed two sets (30 samples each) of synthetic microvessel tubes with distinct orientional structures, see Fig. 3C. We aimed at mimicking the dissimilarity between healthy and post-stroke angiogenesis occurring before and after the induction of thrombotic occlusions on a penetrating artery within a microvascular unit. The first synthetic set contained randomly oriented vascular segments, which one would expect in healthy microvascular networks. On the other hand, samples in the second set were designed to follow radial orientations observed from realistic post-occlusion OCT acquisitions. Random vessel radii ranging from 2–4 $\upmu$m and multiple flow directions were assigned to capillary segments in our synthetic data. We approximated flow and intravascular PO2 using our random forest predictor described in “Methods” section. We extracted an MRI response simulating several gradient directions uniformly distributed in space. These directions are controlled by two separation angles, namely, $\Delta \theta _1$ and $\Delta \theta _2$, see Fig. 3A. We carried our experiments in this subsection after setting both $\Delta \theta _1$ and $\Delta \theta _2$ to 45$^{\circ }$. Our simulations show variations in signal behavior as a result of changing gradients associated with different vascular orientations. An example of simulated output from both randomly- and radially-oriented samples is shown in Fig. 3B. It is clear that after the second diffusion gradient following the $180^{\circ }$ pulse, signals recover at different rates depending on gradient directions. When the direction is perpendicular to the vascular flow, advection of spins had no contribution on signal loss. On the other hand, diffusion gradients that had components aligned with flow direction introduced a noticeable loss in signal intensity. This observation suggests that a quantification of the differences between signal readouts at TE time point can uncover useful information about vascular orientation, and thus be used as a signature of angioarchitecture changes associated with microthrombosis.

### Effect size using different MRI parameters

**Figure 4:**
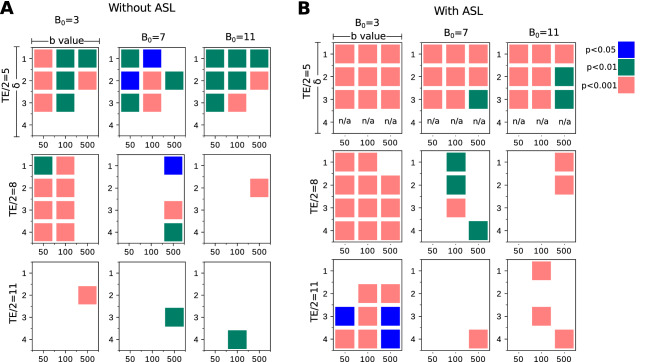
Using a subspace of parameters that determine our diffusion MRI sequence, we plot the corresponding statistical p values computed between our two synthetic groups based on their simulated signal-loss ($\psi$) values. (**A**) Without using ASL. (**B**) With ASL.

To investigate the statistical significance between the randomly- and radially-oriented synthetic samples based on their diffusion-based MRI responses, we quantified the sample-wise maximum difference of signal loss at readout. Let $S_0$ be the signal readout when using no diffusion gradient and $S_i$ be that measured when using a gradient *i* with direction ${\mathbf {u}}_i$. We calculate the maximum difference of signal loss as $\psi =max(\{S_i/S_0\})-min(\{S_i/S_0\})$, where *i* is the index of the gradient directions used, see Fig. 3D. Larger differences imply more anisotropic vascular orientations (i.e., radially structured in our case). We used a non-parametric Mann–Whitney test to examine the statistical significance between $\psi$ values associated with the two synthetic sets. We retained the set of p values calculated after using a subspace of B0, TE, $\delta$ and *b*, see Fig. 4. Gradient directions here are consrtucted using $\Delta \theta _1=\Delta \theta _2=30^{\circ }$. We repeated the experiment with and without eliminating signal contributions of spins in the extravascular space, i.e., using ASL. As anticipated, we observed larger effect sizes, in general, when using ASL. The selection of the TE value had a key role in increasing the difference between the two set of measurements. The lower the TE value, the greater the statistical significance. A comparable effect was observed of the field strength B0: a higher B0 reduced the difference between the two configurations. Furthermore, an increased effect size was observed when using longer gradient time $\delta$. Conversely, the effect associated with the various *b* values on effect size was unnoticeable.

### Diffusion-based MRI signatures of microstrokes using realistic simulations

**Figure 5:**
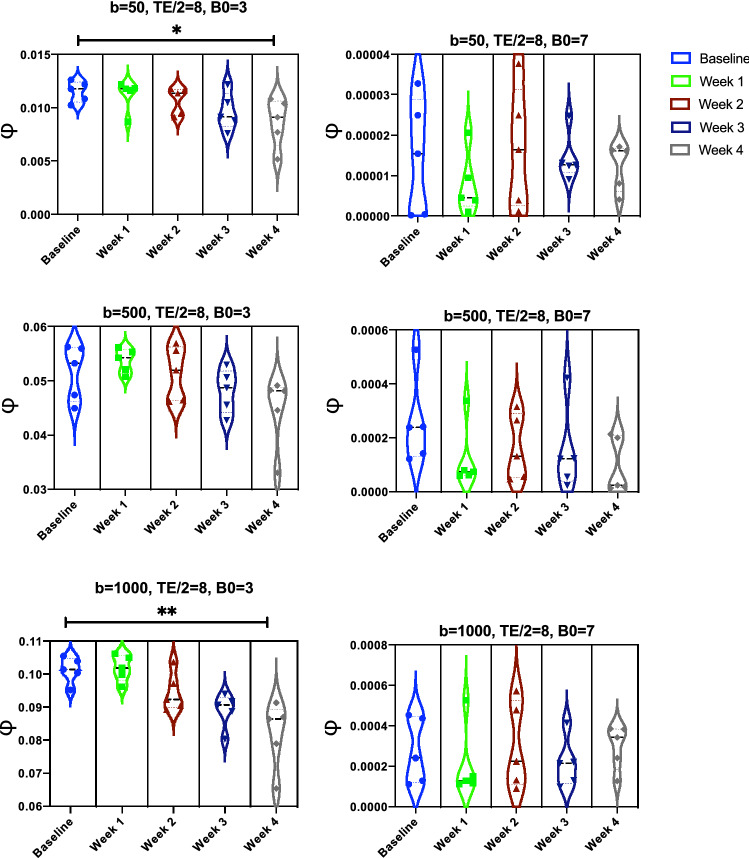
Statistical significance between the ratio of minimal signal loss $\phi$ simulated from our OCT angiograms before and after occlusion through our multi-directional IVIM scheme without involving ASL.

**Figure 6:**
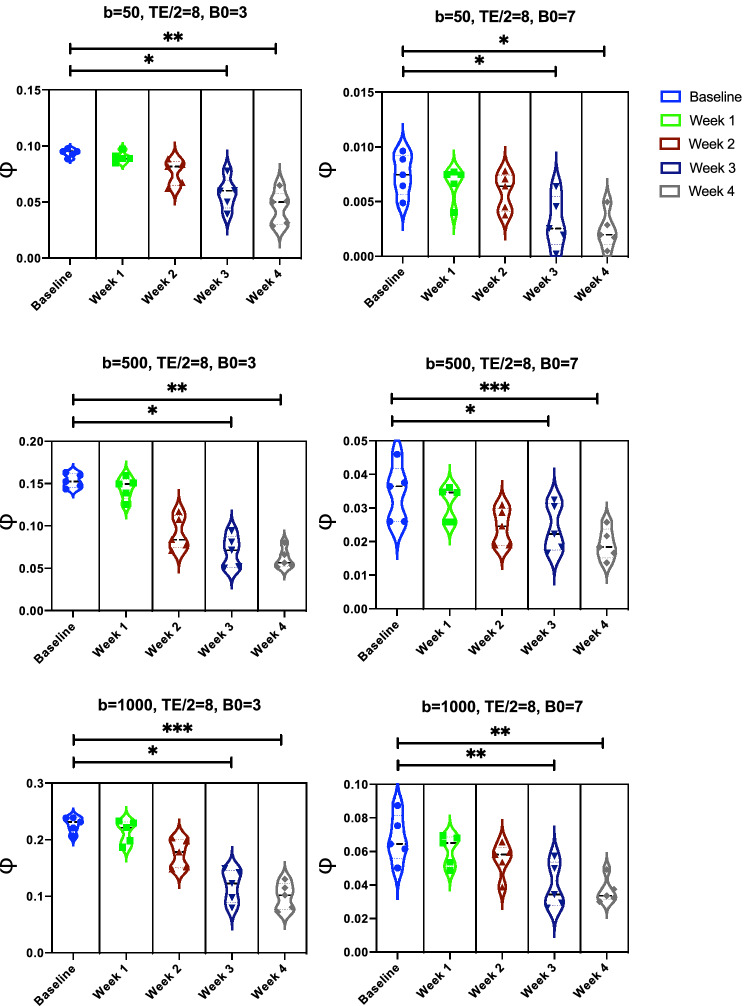
Statistical significance between the ratio of minimal signal loss $\phi$ simulated from our OCT angiograms before and after occlusion through our multi-directional IVIM scheme by involving ASL.

Exploiting our OCT acquisitions of the angioarchitecture acquired longitudinally following photo-thrombosis, we then investigated the capability of the above sequences at detecting a longitudinal change in microvasculature that reflects in vivo conditions. Here, we report the statistical differences, in terms of the ratio of minimal signal loss, $\phi$, between healthy (baseline) and after-lesion (at week 1, 2, 3 and 4) acquisitions. As explained in Fig. 3D, $\phi =1-max(\{S_i/S_0\})$, where *i* is the index of the gradient direction used. We performed our experiments with and without involving ASL, using different sequence parameters, by varying the field strength B0 and the *b*-value while setting TE, $\delta$ and $\Delta$ to 16 ms, 3 ms and 6 ms, respectively. To quantify the values of $\phi$ from each sample, we simulated uniformly distributed gradient directions using $\Delta \theta _1 , \Delta \theta _2 = 30^{\circ }$. Then we carried out the Friedman’s test followed by post-hoc comparisons to study the statistical significance of $\phi$ calculated at the baseline and at the following 4 weeks after occlusion. From Fig. 5, it is observed that no reliable difference was achieved when excluding ASL from our simulations. On the other hand, from Fig. 6, the use of ASL suggests that the proposed marker, $\phi$, is effective in differentiating between samples at baseline and after the 3rd and 4th weeks of occlusion. For example, at B0 = 3T and $b=$500 s/mm$^2$ , $\phi =0.1533 \pm 0.0083$, whereas it is $0.06978 \pm 0.01890$ and $0.06148 \pm 0.01187$ at week 3 and 4, respectively. Noticeably, the scaling of $\phi$ increases with lower B0 field strengths and higher *b* values.

### Robustness of the proposed signature

**Figure 7:**
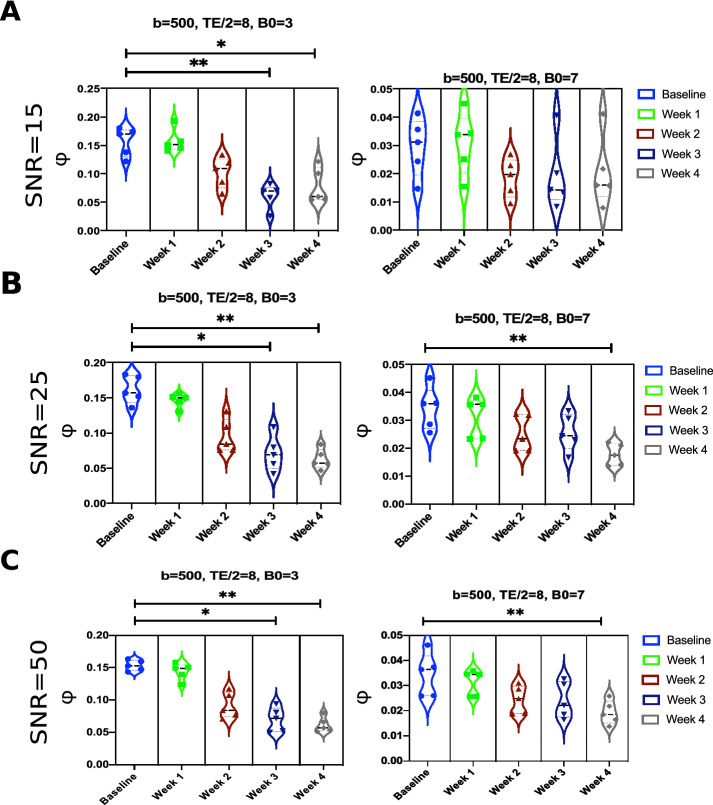
The effect of different SNR, namely, (**A**) SNR = 15, (**B**) SNR = 25, and (**C**) SNR = 50, on the statistical significance in terms of the ratio of minimal signal loss $\phi$. The simulations are carried out on our realistic (OCT) angiograms before and after occlusion using ASL technique.

Here, we investigate the effect of having various levels of signal-to-noise ratio (SNR) on the statistical significance in terms of the simulated $\phi$ values between baseline and after-lesion OCT acquisitions. As previously shown, eliminating ASL from our simulation resulted in no statistical difference of $\phi$ values between the healthy and occluded microvasculature in the majority of cases. Hence, the focus here is centered about the potential signature achieved while using the ASL technique. In Fig. 7, $\phi$ values are calculated at the baseline and at the following 4 weeks after occlusion, at B0 = 3 or 7T, and at 3 different noise levels with SNR = 15, 25 and 50. We set the *b* value to 500 s/mm$^2$ and we set TE, $\delta$ and $\Delta$ to 16 ms, 3 ms and 6 ms, respectively. We carried out the Friedman’s test followed by post-hoc comparisons to study the statistical significance. It is noticed from Fig. 7 that a robust statistical difference between the baseline and 3–4 weeks after occlusion is maintained at a low field strength, B0 = 3T. This observation is persistent at all noise levels. On the other hand, when using B0 = 7T, we noticed no statistical significance when a higher level of noise is introduced in our simulation, at SNR = 15. We noticed that a statistical significance at B0 = 7T is achieved between the baseline and 4 weeks after occlusion only when lower noise levels are involved, at SNR = 25 ,50.

## Discussion

Monte-Carlo MRI simulations have been previously used to quantify the functional MRI origin and to investigate MRI fingerprints of cerebral microvasculature^22,23^. In these previous studies, the reliability of such simulations has been also validated through the comparison with experimental measurements. Here, we leveraged such Monte-Carlo simulations and OCT microscopic imaging to study potential diffusion MRI signatures of microvascular architecture after experimentally inducing photothrombosis. Our results support the hypothesis that such biomarkers are achievable, under certain assumptions, by exploiting the radial arrangement of microvascular capillary compartments post-lesion. Thus this work suggests that quantifying the differences in signal readouts arising from utilizing multiple gradient directions can characterize such vascular orientations. Simulation outputs provided useful insights about the prospective experimental implications. The use of ASL is of critical importance when characterizing microvascular occlusions based on the associated disruption in vascular geometry. In the mouse cerebral cortex, microvascular density sums to less than 0.05 mm$^3$ of a tissue volume of 1 mm; this ratio decreases with depth^24^. Eliminating the MRI signal contributed from the extravascular space is thus necessary. ASL approaches have already shown promising applications in measuring regional cerebral blood flow (rCBF) or perfusion^25–27^, and in assessing vascular disorders^26,28,29^. Our simulation outcomes justify the selection of ASL-coupled multi-directional diffusion MRI used by Well et al.^17^ to annotate flow patterns in the mouse cerebral cortex. We observed a noticeable drop in statistical significance between the responses of healthy and lesioned vasculature at longer echo time TE. This is due to the dominant T2/T2* shortening caused by deoxyhemoglobin distribution, opposed to that rising from diffusion gradients. A similar conclusion of reduced statistical differences was obtained when examining the responses at higher B0 values. Ultra-high field strengths, despite improving SNR, translate into shorter T2* and T2^30,31^. In other words, The increased B0 inhomogeneity at higher B0 leads to more signal loss, especially with longer echo times, which offset the advantage of its higher SNR. A straightforward approach would employ shorter echo time to mitigate the adverse effect of stronger fields^32,33^. This is supplemented through our analysis of the effect of different levels of SNR of the readout signal on the reliability of our microstroke signature. The proposed signature was more susceptible to noise at higher B0 values. Despite using the ASL technique in our analysis, we speculate that the inhomogeneity at higher B0 encoded more effect of large vessels and vascular components that have less contribution to radial orientation of the vasculature. On the other hand, More robust signature has been shown at low field strengths B0 = 3T.

It is known that the capillary component constitutes about 5% of the entire volume of a neurovascular unit^34^. Therefore, the majority of our ASL-free MRI signal is constructed from the extravascular space that cannot encode the microarchitecture of the vasculature. According to the IVIM scheme, there should be a larger signal component accumulated due to the advection process. In an IVIM scheme, very low b values reduces the SNR of the received signal. Such low quality signal cannot be exploited to detach the limited contribution of the radially oriented capillary. Our experiments demonstrates that slightly higher b values but with ASL to eliminate signal contribution from the extravascular space can be used to differentiate random and radially oriented capillary. More comprehensive investigations, e.g., study of fractional anisotropic flow, are conditioned on further improvements of our simulation framework. We employed a machine learning approach to predict PO2 and blood flow distributions across our vascular networks to enable this study. These measurements can be improved through oxygen transport modeling based on more adequate vascular computational graphs. It is to be mentioned that such modeling remains challenging since it requires tedious manual efforts and can be infeasible at scale^22^. It is known that larger microstrokes impose T2 changes in tissue. The proposed modeling could be improved through the incorporation of measured T2 tissue changes to understand their impact with a more realistic simulation. Another aspect of improvement is related to integrating a model of the restricted diffusion in tissue, instead of assuming a constant extravascular T2 field. An improved framework could encompass the magnetic perturbations induced by susceptibility interfaces between vessels and cells, and the permeability of the vessel wall^35^.

## Methods

### Animals

Animal handling was performed in accordance with the ARRIVE guidelines and the recommendations of the Canadian Council on Animal Care. All experiments were performed in accordance with relevant guidelines and regulations. All procedures and handling were also approved by the Animal Research Ethics Committee of the Montreal Heart Institute. Five C57BL/6J male mice of age 3–6 months were used. Cranial window implantation was carried out for each mouse over its left barrel cortex (0.5 mm posterior to bregma, 3.5 mm lateral to the midline) to perform OCT imaging. Following scalp retraction, a craniotomy with a diameter of 3 mm was done using a micro-drill and the dura was kept intact. We covered the exposed brain surface with a stacked four-layer glass cover slip (3 $\times$ 3 mm, 1 $\times$ 5 mm diameter) and sealed it with dental acrylic cement to prevent potential infection. A fixation bar was glued to the skull using the dental acrylic. During surgery, physiological parameters, including electrocardiogram, respiration, heart rate and oxygen saturation of the isoflurane-anesthetized mouse were continuously monitored by a small animal physiological monitoring system (Labeo Technologies Inc. Canada), whose heated platform module also maintained the mouse body temperature at 37 $^\circ$C. OCT acquisitions were performed on awake resting mice to avoid the modulation of vascular and neural physiology^36,37^ by anesthetics. During image acquisition, the mice were placed on a free treadmill wheel with their head fixed on a metal frame by the surgically attached bar. It is to be mentioned that OCT-angiography is phase-sensitive and that even sub-pixel motions can dramatically diminish signal to noise ratio (SNR), and hence it is important that the mice stay still during imaging sessions. Accordingly, we trained the mice for head restraint prior to OCT measurements to habituate them to head fixation and reduce their stress. After a week of training on the treadmill wheel, the mice were able to reach a resting state within five minutes after being fixed onto the setup. They were able to stay calm and still for periods of minutes separated by short bouts of locomotion. After the initial baseline measurement, the mice were still trained every day between imaging sessions to maintain their habituation to head restraint throughout the study. The mice were closely monitored for locomotion during image acquisitions.

### Ischemic stroke model

The stroke model exploited a localized photo-thrombosis procedure which is based on a photochemical reaction introduced by Watson et al.^38^. Mice were first intraperitoneally administered Rose Bengal (15 mg/ml, 0.2 ml), a photosensitive dye. A selected cortical region, free of large vessels, was irradiated by a focused green laser beam, since large-vessel thrombosis could lead to a less predictable and less controlled outcome. In addition, avoiding regions with large pial vessels could also minimize the effect of tail artifacts in OCT angiography images^39^. Green light illumination enforces Rose Bengal to produce free radicals that lead to a damage in the endothelium of the microvasculature, thereby triggering discoid platelet aggregation that eventually leads into thrombotic occlusions. The whole process of photo-thrombosis was managed and monitored using a home-built laser speckle imaging system.

### OCT acquisition system

Imaging of cortical structure and vasculature was performed with a home-build spectral-domain OCT. A broadband light source centered at 1310 nm from a superluminescent diode (SLD) (LS2000C, Thorlabs, USA) was split between the sample arm and the reference arm by a 90:10 fiber optic coupler (TW1300R2A2, Thorlabs, USA). A long working distance objective (M Plan Apo NIR 10X, Mitutoyo, Japan) was installed at the end of the sample arm to focus the collimated light beam into the tissue sample. The spectral interferogram was registered by a spectrometer (Cobra 1300-[1235–1385 nm], Wasatch Photonics, USA) and then digitized by a frame grabber (PCIe-1433, National Instruments, USA). Dispersion mismatch between the two arms was first carefully compensated with N-SF11 compensation glass (Edmund Optics, USA), and the small residual mismatch was then finely corrected with a numerical compensation technique^40^. The axial resolution was measured to be about 4.15 $\upmu$m in biological tissues. The lateral resolution in tissue was about 2.3 $\upmu$m. In the sample arm, a dichroic filter was placed to transmit the infrared light used by the OCT system and deflect the visible light for wide-field imaging. The wide-field imaging helped locate the region of interest (ROI) to be scanned by OCT. The sample arm consisted of a galvanometer scanner, a beam expander and an objective lens. The arm was mounted on a motorized vertical translation stage (MLJ150/M, Thorlabs, USA). Adjusting the depth of the imaging focal point can be performed by elevating or lowering the vertical stage. The treadmill wheel onto which the mouse was attached was fixed on a motorized XY linear translation stage (T-LSR, Zaber Technologies, Canada) for fine adjustment of the relative lateral position of the cranial window with respect to the light beam. The 3-axis motion control was integrated into our acquisition software.

### OCT scanning

We scanned a 1 mm $\times$ 1 mm region with the photo-thrombosis-induced lesion located in the center. Our volumetric scans of the cortex contained 450 B-frames, each of which was composed of 500 A-lines. First, raw spectra were resampled in k-space and then multiplied by a Hanning window. Then, inverse Fourier transform (IFT) was applied to obtain 3D complex-valued OCT structural images. B-scans were repeated twice at each position along the slow axis. Global phase fluctuations (GPF) caused by sub-pixel motion within repeated B-frames were corrected based on the assumption that dynamic tissue only accounts for a very small percentage of brain tissue and that phase and intensity of light reflected from static tissue remain constant^41^. In principle, light reflected by moving red blood cells (RBC) experiences a large phase shift and/or a big intensity change. Thus, we obtain a vascular image by taking the phase and intensity difference between GPF-corrected repeated B-frame^42^. The resulting 3D angiograms were filtered with a 3D Gaussian smoothing kernel with a standard deviation equal to 1 pixel in all three dimensions. A fast axis scan rate of 90 Hz was used, which resulted in an acquisition time of 10 s per volumetric scan.

### OCT reconstruction

In order to extract more comprehensive information of the capillary network in the cortex, three time-resolved OCT-angiographies were performed in the same ROI with light beam being focused at three different cortical depths, namely 250 $\upmu$m, 400 $\upmu$m and 550 $\upmu$m beneath the cortical surface. To achieve a shift of the axial focus in the tissue, we preformed a vertical translation of the objective lens in the sample arm mounted on the vertical translation stage. After, OCT stacks for each animal that were taken considering three different depth-dependent setups, are recombined to form one stack. To obtain the final desired stack, we followed a procedure based on measuring the mean of local entropies computed from each stack after normalizing and convolving it with a set of Gabor filters. Mean entropy measures for all the stacks are then normalized with a softmax function imposed on the axis representing the index of each stack. Voxel intensities in each stack are then weighted by the corresponding normalized local entropies. Finally, we take the sum of the weighted intensities from all the stacks to reconstruct the output stack. In our procedure, we used 18 two-dimensional Gabor filters built with orientations $\in {0, \pi /6, \pi /3, \pi /2, 2\pi /3, \pi }$, phase offset $\in {2.5, 5, 7.5}$ and a wavelength = 0.01. The kernel used for calculating the local entropy is of size (15, 15). We ran slice-wise calculations to quantify the entropy of each stack. In our chronic microstroke study, we performed 6 OCT imaging sessions over 28 days. The baseline measurement was taken one day before photo-thrombosis. The second imaging session took place 3 days after the thrombotic lesion was induced in the mouse brain, and the following 4 measurements were made 8 days, 14 days, 21 days and 28 days respectively after the ischemic stroke event.

### Vascular segmentation and graphing

Many works on vessel segmentation have been presented in the literature. The best recent schemes were based on U-Net neural networks^43^ with their convolutional architecture that accepts images of arbitrary sizes. In this work, we used the LadderNet architecture employed in^44^, which is inspired from the U-Net one but with more interconnected information paths. This architecture can be seen as multiple stacked U-Nets with more pathways. Compared to the conventional U-Net scheme, the shared-weights structure in the LadderNet allows horizontal propagation through the stacked U-Nets which forces the learning process to be made in earlier layers, and thus provides better results^19^. We trained and evaluated our network on an in-house prepared and annotated dataset from two-photon microscopy angiograms. This two-photon microscopy dataset was prepared and used in previous studies^20,21^. It is to be mentioned that manually annotating our OCT angiograms is infeasible due to its large size. Therefore, we used our trained network to automatically annotate them. The two-photon microscopy dataset used for training consisted of 59 8-bits 2D grayscale images of 256 $\times$ 256 pixels which were then split into 70%, 15% and 15% portions for training, validation and testing, respectively. Images were standardized by subtracting the mean and dividing by the standard deviation. Contrast limited adaptive histogram equalization was then applied to correct light imbalance. Images were adjusted with a gamma value of 1.2. Training, testing and validation processes were done patch-wise (patch size of 32 $\times$ 32) after augmentation with random rotations. Each of the down and up streams paths in the network had 4 convolutional layers. A leaning rate of 0.001 was used. We applied the trained network on each slice in our 3D OCT angiograms to obtain the corresponding delineated vascular structures. After segmentation, we employed a graphing method, proposed recently by Damseh et al.^21^, to transform our binarized inputs into a fully-connected graph-based skeletons. The method initiates geometric grid graphs encapsulated within vascular boundaries and iteratively deforms them toward vessel centerlines. Converged geometries are then refined and converted into graph-based skeletons. The output graph models consisted of nodes distributed along vessel centerlines to capture the geometry and edges connecting these nodes to represent the inherited topology. The method assigns vessel radii to graph nodes with unique identifiers to each set of them located at a certain vascular branch/segment. We used the VascGraph toolbox associated with that work, available on https://github.com/Damseh/VascularGraph, to generate and visualize the anatomical graphs.

### Flow and PO2 regression

To prepare the generated anatomical model to undergo MRI simulation experiments, we had to assign biophysical quantities across vascular compartments, namely flow and PO2 values. Flow values are essential to guide the advection process in our MRI simulation framework. On the other hand, PO2 values are necessary for reconstructing the magnetic field perturbations, which drives the T2* effect in our MRI simulations. The procedure of computing these perturbations from PO2 is explained later in this section. Resolving flow and PO2 values through biophysical simulations is tedious due to the low-quality nature of OCT inputs, which hinders the formulation of a correct geometric domain suitable for such computations. In other words, such computations are extremely sensitive to topological errors that can easily disrupt the final solution. Here, instead, we followed a machine-learning based approach that utilizes an ensemble of random decision trees, i.e., random forests, fitted on experimental data collected from mice models in a previous work performed by Moeini et al.^34^. The data consisted of $>300$ measurements of PO2 and Flow values of cerebral microvascular segments having different sizes, types and located at different cortical depths. We built two separate random forest models that accept radius, type and depth information for a vascular segment and output the corresponding flow and PO2 values. Types of vessel segments were determined after thresholding on the radius value of 3 $\upmu$m^34^. Vascular segments with radii above the threshold were randomly set to be either arteries or veins, whereas the rest were set as capillaries. Following this procedure, we only needed an approximation of the vascular geometry to capture the anatomical features required by the random forest model, and avoided the dependency on the biophysical modeling that requires accurate annotations and is highly prone to any topological error induced in the vascular model. Each of the two random forests consisted of 100 decision trees with 10 maximum depth. The architecture of our regression forests was selected after assessing the mean square error (MSE) resulting from using different architectural setups. The same measure of MSE was used to determine the quality of a split in the single decision trees that compose the forest model. The minimum number of samples required to split in each decision tree has been set to 2. Bootstrapping was used to reduce the variance resulting from the outputs of multiple trees fitted on random sub-samples, with replacement, from the original training set^45^.

### MRI simulations

There have been several Monte-Carlo frameworks built to study the diffusion MRI response^22,23,46,47^. In our study, we implemented a Monte-Carlo MRI simulation code in Python following the same routines of previous protocols^22,23^, which was built using Matlab. Simulation principles in these works were originally based on descriptions detailed in^46^. The previous implementations were performed to tackle the Blood-oxygen-level-dependent (BOLD) MRI response. Here, to align with our study goals, we developed new functionalities to create multi-directional spin echo DWI sequence simulations. In our numerical MRI trails (coinciding with the aforementioned studies), it was assumed that the MRI response in the mouse brain could be approximated by probing the behavior of a large number of hydrogen water protons moving in background due to free diffusion and constrained advection processes. Practically, our assumption incorporated the following in-detail descriptions: T2 value in tissue regions is fixed but varies within the vascular space; T2* effect has spatial variations subjected to the level of deoxyhemoglobin within the vasculature, see Fig. 2A. As suggested in previous works designed for studying vascular-based MRI responses^22,23,48,49^, we neglected a set of factors that have insignificant effect on the extracted signal: hydrogen atoms not bound to water, additional iron presence in basal ganglia and some other brain parts, external B0 and B1 fields inhomogeneities, gradient nonlinearity, hematocrit variability. Also, to enable our analysis, we excluded the macroscopic magnetic field destruction occurring due to imperfect shimming or the microscopic field differences emerging from the applied gradients.

In our Monte-Carlo simulations, we measured the voxel-wise response based on summing the accumulated complex phase from $1 \times 10^{6}$ spins, after initiating their positions at $t=0$ using a uniform probability distribution that covers the spatial domain, and then updating their location every $\Delta t=0.05$ ms. The protons were let to diffuse isotropically with a diffusivity constant of 0.8 $\upmu$m$^2$/ms^50^. We also update intravascular proton locations at each time step to account for the advection process that is controlled by the predicted velocity/flow maps. With the assumptions mentioned above, among the relaxation constants necessary for our computations is that attributed to the local magnetic field inhomogeneities, affecting T2*, due to the presence of deoxyhemoglobin in the vasculature. Another important relaxation factor affecting T2 is that related to the spin-spin coupling, where it is computed based on the fitted models illustrated in^51^.

In an IVIM scheme, assuming a negligible exchange between the IV and EV components during the echo time^17^, the total DW voxel signal captured at a gradient *i*, with its direction $\mathbf{u} _i \in \mathbf{U} = \{ \mathbf{u }_1, \mathbf{u} _2, \ldots \mathbf{u} _n\}$, could be expressed as:

1 $$\begin{aligned} S_i=S_0 [fe^{-b_{i}D_{i}^*} +(1-f)e^{-b_{i}D_{i}}] \end{aligned}$$

where $S_0$ is the response at zero diffusion gradient, *f* is the fraction of vasculature in the voxel, and $b_i$(s/mm$^2$) is a parameter that characterizes the diffusion gradient *i*. The term $D_i$ is the apparent diffusion coefficient (ADC) along a given direction and it is influenced by the Gaussian diffusion in tissue. On the other hand, $D^*_i$ is the pseudo-diffusion coefficient (PDC) representing the perfusion of spins in the microvascular network. A diffusion gradient consists of two short pulses of duration $\delta$, with amplitude *G*, separated by a short amount of time $\Delta$. The corresponding *b* value can be obtained as:

2 $$\begin{aligned} b = \gamma ^2 G^2 \delta ^2 \left( \Delta - \frac{\delta }{3} \right) \end{aligned}$$

where $\gamma$ is the proton gyromagnetic ratio of hydrogen. The formula in (1) could be regarded as a standard IVIM model for the diffusion signal at readout assuming that both tissue and blood have the same T2. Here, our simulations aim at more realistic outputs by taking into account the interactions of individual spins with the T2 map and the magnetic perturbations varying in the intra- and extra-vascular spaces. Our simulation framework approximates the diffusion signal by cumulatively averaging the phase shift of the initiated spins incurred due to their diffusion and advection over time. We start our simulation by applying the 90$^\circ$ pulse resulting in a unity signal amplitude for each spin, and thus an average signal equal to 1. At each time step *n* and for each spin, we update the *x*, *y*, *z* coordinates of each spin based on the diffusion and advection effects at their present location. We then diminish the signal amplitude of each spin due to T2 relaxation and update their phase shift due to field perturbations. The updated signal amplitude of each spin based on $\Delta t$ time shift and after *n* time steps is given as follows^22,23^

3 $$\begin{aligned} s(n)=\left| e^{\alpha _n + \beta _n} \right| \end{aligned}$$

where the terms $\alpha _n$ and $\beta _n$ are calculated respectively as

4 $$\begin{aligned} \alpha _n= \alpha _{n-1}-\left[ \frac{\Delta t}{\text {T2}_{x,y,z}}\right] , \ni \alpha _0=0 , \end{aligned}$$

5 $$\begin{aligned} \beta _n= \beta _{n-1} + j d \gamma \Delta t \left[ \Delta \text {B}_{x,y,z} + \text {G}_{x,y,z} \right] , \ni \beta _0=0 \end{aligned}$$

The constant *j* is the imaginary number $\sqrt{-1}$; $d=1$ before the 180$^\circ$ pulse in our DWI sequence whereas $d=-1$ after it. The terms $\Delta \text {B}_{x,y,z}$ and $\text {G}_{x,y,z}$ are magnetic field perturbations at the *x*, *y*, *z* coordinates from deoxyhemoglobin in the vasculature and the applied gradient field, respectively. We followed the numerical method described in^52^ to compute the perturbations in the magnetic field, $\Delta \text {B}_{x,y,z}$, by convolving the susceptibility shift volume, which is calculated depending on the level of PO2, with an ellipsoidal kernel oriented with the field B0. In (4), T2$_{x,y,z}$ in the extravascular space is spatially fixed and obtained in seconds as

6 $$\begin{aligned} \text {T2}^{(tissue)}_{x,y,z}= \left[ 1.74*\text {B0} + 7.77 \right] ^{-1} \end{aligned}$$

On the other hand, this parameter spatially varies in the vasculature depending on the oxygen saturation level (SO2)^51^:

7 $$\begin{aligned} T2^{(vessel)}_{x,y,z}=\left[ 12.67*\text {B0}^2*(1- \text {SO2}_{x,y,z})^2 + 2.74* \text {B0} - 0.6 \right] ^{-1} \end{aligned}$$

The SO2$_{x,y,z}$ values were calculated from their PO2$_{x,y,z}$ counterparts, predicted through our random forest scheme as previously explained, based on the Hill conversion equation with coefficients specific for C57BL/6 mice (h = 2.59 and P50 = 40.2)^53^. Hematocrit values necessary to compute the field susceptibility $\Delta \text {B}_{x,y,z}$ as described in^52^ were assumed to be 0.44 in arterioles and venules, and 0.33 in capillaries^54^.

In the task of finding a signature about the deterioration in the microvascular architecture occurring due ischemic thrombotic events, our interest is the set of ratios $R = \{ r_i = \frac{S_i}{S_0}, i \in \{1, 2, \ldots , n \} \}$, calculated through applying *n* gradients with directions equally sampled from all possible choices in the 3D space. As discussed in “Introduction” section, previous works^8,9^ reported that voxels with thrombotic ischemia exhibit a highly radial orientation of microvessels in the lateral plane around the lesion core. This implies that a higher perfusion rate, i.e., an increased loss in MRI signal, would be observed when a gradient field is parallel to the lateral plane compared to that occurring when it is perpendicular to it. In general, healthy voxels beneath the pial surface are assumed to be composed of randomly oriented capillary segments, and thus would result in comparable MRI signal loss regardless to the direction of the selected gradient. Hence, the ratio $\phi = 1-max(R)$ could be regarded as a biological marker distinguishing healthy voxels from those having a thrombotic lesion, see Fig. 3D. Beside computing $\phi$ through probing spins behavior in an entire voxel or ROI, we also studied the response when neglecting the extravascular spins, thus omitting the diffusion term in (1). Such procedure could be practically translated with ASL. The ASL technique is integrated in our simulation framework through simply constructing the initial nuclear spins to be only contained within the vascular space. These spins are then submitted to the effect of diffusion and advection, and their signal is updated as previously described.
